# A Pre-Clinical Study of Sub-Anesthetic Ketamine as Remedy in 5-Fluorouracil-Induced Cachexia Model

**DOI:** 10.3390/life13010008

**Published:** 2022-12-20

**Authors:** James Wang, Zen-Cheng Lin, Brian Bor-Chun Weng

**Affiliations:** 1 Astromedical Biotechnology, LLC, New Taipei City 25147, Taiwan; 2 Department of Microbiology, Immunology, and Biopharmaceuticals, College of Life Sciences, National Chiayi University, Chiayi 60004, Taiwan

**Keywords:** cachexia, chemotherapy, 5-fluorouracil, ketamine, animal modeling

## Abstract

Around 0.5–1% of the world population is suffering from cachexia. In particular, cancer patients under cancer radio-chemotherapy have a high prevalence of cachexia, especially during the end stages of therapeutic treatment. Clinically, chemotherapeutic 5-fluorouracil (5-Fu) treatment often leads to the development of adverse effects, such as leukopenia, immune dysfunction, anorexia, muscle wasting, etc., and 5-Fu also tends to exacerbate the occurrence of cancer cachexia. Currently, there are very limited drug choices when seeking to revive cachexia patient’s health quality while enduring a full therapeutic regimen as part of advanced cancer therapy. The present study employed chemotherapeutic drug 5-Fu-induced cachexia-like conditions in Balb/c mice. After 8 days of 5-Fu treatment, mice had begun to show cachexia-like symptoms such as weight loss and reduced food intake. After one day of washing out, the cachexia animals received a single dose of either saline solution as a mock dose or a low dose (15 mg/kg BW) or high dose (30 mg/kg BW) of ketamine at day 10. For the following 7 days, food intake, body weight, and mortality were monitored. Data were analyzed with the LOCF (last observation carried forward) method. Improved survival rates were obtained in ketamine groups. Ketamine administration at the high dose of 30 mg/kg BW demonstrated effectively diminished weight loss due to cachexia, and also successfully improved overall survival. The current study demonstrates that a sub-anesthetic level of ketamine administration supports overall beneficial outcomes in 5-Fu-induced cachexia and outlook as a potential clinical remedy.

## 1. Introduction

Cachexia commonly happens during the end-stage treatment of many illnesses, such as cancer, heart failure, chronic obstructive pulmonary disease, liver failure, kidney failure, stroke, rheumatoid arthritis, severe burn injury, and HIV/AIDS [1]. The prevalence of cachexia was reported to be higher in older cancer patients in a clinical multivariate analysis survey study, with significant correlations to factors such as risk of depression, low food intake, types of cancers, etc. [2]. However, currently there are no approved drugs that effectively treat cachexia, and poor diagnosis with insufficient clinical criteria and difficulties in animal-modeling the disease have greatly hindered the development of remedies. It has been reported that the chemotherapeutic drug 5-fluorouracil (5-Fu) exacerbated skeletal muscle loss in mouse models [3,4]. Moreover, Farhang-Sardroodi et al. [5] have also demonstrated with a mathematical model that simulated 5-Fu dosing levels and schedules in intention to minimize muscle loss due to cachexia during cancer chemotherapy.

Weight loss is a typical symptom of cachexia and is often associated with anorexia, chronic inflammation, insulin resistance, malabsorption, and protein breakdown. Naito T. [6] reviewed some randomized controlled trials that treated cancer cachexia using corticosteroids, progestins, nonsteroidal anti-inflammatory drugs (NSAIDs), thalidomide, and eicosapentaenoic acids (EPA) with different regimes and saw reasonable benefits. In addition, some novel agents are currently under phase 3 randomized controlled trials, including the ghrelin receptor agonist (anamorelin hydrochloride), the anti-IL-1alpha monoclonal antibody (MABp1), and a nonsteroidal selective androgen receptor modulator (enobosarm). Certainly, herbal extract-derived bioactive compounds developed to control inflammation have also shown some promises.

Ketamine is a compound with an expired patent license that was originally created for anesthetic purposes. Lately, it has been under intensive study in the development of old-drug-new-use applications for the treatment of anxiety and depression. Mechanistic actions of ketamine have been reported, such as its role as an NMDA (N-Methyl-D-aspartate) receptor antagonist, a DOPA (dopamine) transporter inhibitor, and a promoter of 5-HT (5-hydroxytryptamine) release [7]. With recognitions of all the above mentioned receptors are also presenting in diverse immune cell populations. Ketamine exhibits a role in the modulation of immune functions. Administration at a sub-anesthetic level before induction of anesthesia in abdominal surgery patients showed that ketamine was able to attenuate IL-6 and TNF-α production while retaining the cytotoxic activity of IL-2 activated natural killer cells [8]. Furthermore, ketamine, as a noncompetitive NMDA receptor antagonist in modulating neurochemical–immune interactions, exerts anti-inflammatory properties by downregulating the transcription factors NFκB (nuclear factor-kappa B) and AP-1 (activator protein-1) [9]. Indeed, our unpublished pilot data indicated elevated neutrophil to lymphocyte ratio (NLR) in an in situ mammary tumor-bearing mice model, which could be attenuated by i.p. sub-anesthetic ketamine. Previously, in an in vitro study, it was reported that the adhesion of neutrophil to endothelial cells was depressed by ketamine, and the suppression of oxygen free radical generation was also evidenced [10]. 

Ketamine exhibiting remedial advantages in anxiety and depression while demonstrating anti-inflammatory and immunomodulatory properties suggests its theoretical potency in the treatment of cachexia. Cachexia requires clearly defined clinical criteria, which leads to difficulties in establishing fully simulated animal model. Additionally, the chemotherapeutic drug 5-fluorouracil has been known to interfere with DNA synthesis, which may induce cachexia like conditions. The model used in the present study has therefore been established for assessment of the remedial potential of a sub-anesthetic level of ketamine. The promising outcomes have led to patents granted in the USA and Taiwan in 2022, and further endorsements are foreseen in the other 16 countries.

## 2. Materials and Methods

### 2.1. Animals

The pre-clinical animal study (Protocol No. 107017) was approved by the Animal Care and Use Committee of the National Chiayi University, Chiayi, Taiwan. A total of 12 healthy 8-week old male Balb/c mice were acquired from BioLASCO Co., Ltd., Taiwan, and were raised in an environmentally controlled (12:12 dark–light cycle with a constant room temperature of 25 ± 1 °C) specific pathogen free facility. Mice were initially weighed and the 4 animals with lowest body weight were purposely assigned into the normal control group, while the remaining heavier animals were randomly assigned into 4 groups: 1 group for the chemotherapy agent 5-fluorouracil (5-Fu), and 3 for the ketamine-treated groups (mock group (saline, 0 mg/kg BW), low-dose (15 mg/kg BW), and high-dose (30 mg/kg BW)). Each group had 4 animals in one standard plastic cage fed ad libitum. For animal welfare considerations of cachexia condition, each cage had a water bottle with a stainless cap and extended tip (closer to floor). In addition, standard rodent chow LabDiet™ 5001 was smashed into loose particles and provided in a shallow glass dish on the floor close to the tip of the water bottle. Individual body weight, group feed, and water consumption were measured every morning. 

### 2.2. Regimen of 5-Fu Induced Cachexia-like Conditions 

**Scheme 1:**
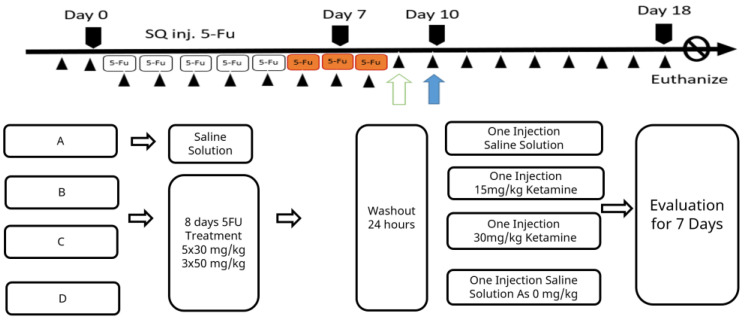
Schematic time schedule for the experiment.

Except for the animals in the normal control group kept for background procedure checking, all other animals were subjected to daily subcutaneous injection (SQ inj.) of 100 μL of 5-fluorouracil (5-Fu™, F6627; Sigma, St. Louis, MO, USA). A daily dose of 25 mg/kg BW 5-Fu was administered for 5 days following a dose of 50 mg/kg BW that was administered for 3 days. After the 5-Fu induction treatments, the therapeutic ketamine counter measure was initiated and data were collected for 7 days until the humane endpoint. To comply with animal ethics and experiences from pilot studies, animal body weight was not allowed to decrease by more than 20% of the original body weight within a week of this regimen being applied. Minor distresses such as avoidance, reduced movement, and increased corner resting time, etc., were monitored. Diarrhea might occur and was apparent as feces stains on hairs around the anus, and this was considered a severe sign of animals being moribund. Specific care was provided with water moisture-softened feed. Individual water and feed consumptions were not recorded during specific care. The experimental timeline is shown in Scheme 1.

### 2.3. Ketamine Administration

Ketamine hydrochloride injection (Ketaset™, Zoetis, Parsippany-Troy Hills, NJ, USA) solution was diluted with intravenous saline drip solution. The mock group received a dose of 100 μL saline, while the low-dose and high-dose ketamine groups received 15 or 30 mg/kg BW of ketamine, respectively. The single dose treatment was administered by intraperitoneal injection on the next day of washout after the completion of the 5-Fu induction regimen. Body weight and water and feed consumptions were measured daily with a digital scale (Sartorius, Göttingen, Germany). At the end of 7 days of data collection, animals were euthanized via CO_2_ overdose. Subsequently, blood samples were collected via heart puncture in EDTA tubes for hematocrit tests and blood work. Immune organs were harvested for other studies.

### 2.4. Statistical Analysis

The statistical analyses were performed on last observation carried forward (LOCF) populations. No statistical inference was made in this observational study. The sample size was estimated (N = 4 per group) based on the best available medical and ethical judgement. In the LOCF populations, all subjects were randomized and treated in the assigned treatment groups. When subjects died before the end of the study, the subject’s last observed data were used for all subsequent observation points.

## 3. Results

Pharmacological intervention with ketamine has not been proposed for the treatment of cachexia. The results indicated that ketamine was effective in slowing down weight loss due to cachexia, and ketamine was also an effective agent in improving overall survival. As shown in Table 1, The mock 0 mg/kg group had a reduction in body weight to 18.8 ± 1.4 g from the baseline (day 10) weight of 22.7 ± 0.5 g at the end of the study (day 17), with a loss of 4.0 ± 1.3 g or −17.4 ± 6.0% of the baseline weight. The low-dose 15 mg/kg ketamine group had a reduction in body weight to 18.3 ± 3.8 g from the baseline (day 10) weight of 22.1 ± 0.9 g at the end of the study (day 17), with a loss of 3.8 ± 2.9 g or −17.7 ± 13.8% of the baseline weight. The high-dose 30 mg/kg ketamine group had a reduction in body weight to 20.3 ± 1.8 g from the baseline (day 10) weight of 22.1 ± 0.7 g at the end of study (day 17), with a loss of 1.8 ± 1.3 g or −8.2 ± 5.9% of the baseline weight. The control group had a reduction in body weight to 21.0 ± 0.6 g from the baseline (day 10) weight of 21.9 ± 0.6 g at the end of study (day 17), with a loss of −0.8 ± 0.6 g or −3.8 ± 2.7% of the baseline weight.

**Figure 1:**
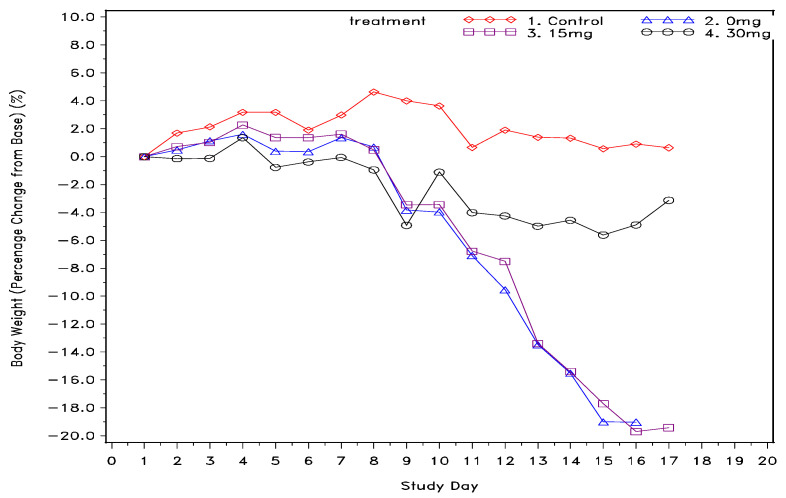
Body weight percentage change from baseline (g) across the entire study post cachexia induction with 5-fluorouracil.

Single dose ketamine treatment was also an effective agent in improving overall survival of cachexia mice. As demonstrated in Table 1, mice in both the control group and 30 mg/kg group were completely survived at the end of study, whereas only 25% of mice in the 0 mg/kg and 15 mg/kg groups were overcome. In addition, as also shown in Table 1, food consumption over time for the high-dose and low-dose ketamine groups had mean values of 16.8 ± 5.7 and 16.5 ± 8.4 gm, respectively, showing a higher feed consumption trend compared to the mock group (mean = 14.6 ± 7.5 gm). Moreover, average water consumption in the high-dose ketamine group was the highest among the 5-Fu-treated groups. Nevertheless, the low-dose ketamine group had less average water intake than the mock group. Furthermore, body weight percentage change over time as shown in Figure 1. revealed single high-dose ketamine injection on day 10 had a body weight rebound and remaining steady in the following days till the end of study. On the other hand, the mock and low dose ketamine groups have continuously losing body weight over the 7 days monitoring period, and about 20% loss in body weight at day 15 to 17 as compared with the basis of day 1. 

## 4. Discussion

Cachexia is characterized by progressive muscle wasting, body weight loss, weakening, and a reduction in physical activity. Anorexia, inflammation, dysfunctions in glucose utilization, and increasing muscle protein breakdown are the key factors in cachexia associated with cancer chemotherapy. Cachexia significantly compromises successful treatment rates in chemotherapy patients and contributes to poor prognosis. It is challenging to simulate cachexia in animal models due to the nature of complications caused by different cancer types, anti-cancer immunity, regimens of chemotherapy, and combinations of other diseases. Moreover, for the treatment of cachexia, some drugs are currently being used or under clinical trial; however, none of them have satisfactory outcomes regarding improved quality of life in cancer patients [1]. Despite clinical evidences suggesting that chemotherapy induces cachexia, the mechanism is still poorly understood, though there is some support from a drug toxicity perspective. It has also been proposed that cancer cachexia is different from chemotherapy-induced cachexia [11]. Indeed, cancer may results in more complicated inflammatory symptoms and immune dysfunctions with altered neutrophil to lymphocyte ratio [4]. Nevertheless, Murphy et al., 2022 shows that 5-Fu chemotherapy in tumor bearing mice significantly exacerbate the loss of skeletal muscle by a dysregulation of microRNAs, and silence of microRNA dependent ERK2 inhibition can prevent 5-Fu induced muscle atrophy [3]. It is worth to note that their 5-Fu (100 mg/kg BW) administrated 3 days apart for 3 times is responsible for the changes of microRNAs but not the cancer itself. It will be interesting to know if 5-Fu alone and the therapeutic regimen differences may contribute to a similar outcome.

Currently, ketamine is intensively being reviewed for new use as a treatment for depression. Whether it may aid psychological status in 5-Fu-induced cachectic mice requires further elucidation. It has been reported that two doses of ketamine (20 mg/kg) at 4 h apart reversed the rat depression model in forced swim tests [12]. Additionally, during the two months of the experiment, no drug-seeking behaviors were observed. Indeed, a recent study demonstrated a dual action of ketamine in regulating both dopamine and GABA-mediating neural signaling, which minimizes its addictive possibility [13]. This evidence reinforces the new uses of ketamine as a cure for critical diseases such as depression and cachexia without worry of addiction. 

Whether ketamine affects appetite in humans remains inconclusive. Nevertheless, appetite seems to either not be affected or never to become a serious issue in animal clinics when ketamine is used to temporarily sedate or surgically anesthetize, which suggests ketamine may have little impact on appetite [12,14]. However, the low food and water consumption observed in mice in the mock and low-dose groups of this study indicates that one of the major factors of cachexia is loss of appetite during chemotherapy, which thus impairs nutritional status and debilitates muscles and adipose tissues atrophy. In the current study, a high dose of ketamine (30 mg/kg) prevented 5-Fu-induced body weight loss while improving feed intake and water consumption over time. This finding regarding ketamine increasing feed intake is in agreement with Chen et al. [14], who reported that a single injection of ketamine (two doses tested at 3 and 30 mg/kg i.p.) improved body weight and food consumption in a mouse activity-based anorexia model. 

Finally, ketamine was assessed in its immunomodulatory functions during immune rejuvenating post radio/chemotherapy induced leukopenia models, together some other potential herbal extracts and generic drugs were assessed in our sibling projects. In those projects, neutrophil to lymphocyte ratio (NLR) was closely monitored. Our preliminary data gathered from limited number of mice indicate that the blood neutrophils population is likely attenuated by ketamine, which attenuate the elevating NLR along with tumor progression in syngeneic mammary adenocarcinoma in both in situ and metastatic models. Furthermore, despite anti-cancer therapy not being a covariate, Barker et al. [13] demonstrated that elevated NLR is associated with systemic inflammation that can be attributed to weight loss and cachexia in cancer patients. Ketamine exhibiting an immunomodulatory role shall be gaining more attention in studies seeking to control inflammation. Indeed, sub-anesthetic doses of ketamine administered i.v. prior to surgical operation have been shown to diminish pro-inflammatory cytokines IL-6 and TNF-alpha for 24 to 72 h after operation [8]. While considering the use of ketamine as an antidepressant, Szalach et al. [15] have also revealed immunomodulatory actions of ketamine which specifically increase regulatory T cell activity and decrease neutrophil activation and ROS production. The immunomodulatory property of ketamine may play a critical role in alleviating cachexia conditions.

## 5. Conclusions

In conclusion, the results of this study support the remedial potential of ketamine in cachexia. Single sub-anesthetic doses of ketamine exhibit promising outcomes in terms of ameliorating cachexia-like conditions in mice when induced by the chemotherapeutic drug 5-fluorouracil. Immunomodulation, muscle regeneration, emotional soothing, and multiple other potential mechanisms may further support the use of ketamine as a treatment for cachexia, which is of importance for future research and its therapeutic application as a remedy to treat cachexia in improving the quality of life of cancer chemotherapy patients.

## 6. Patents

The proposed conclusion has been granted a US patent on 2 August 2022 with a patent number 11400060 issued by the United States Patent and Trademark Office. Additionally, a Taiwanese patent has also been recently approved with the publicized number 202135788, and patent number I786498.

## Figures and Tables

**Table 1:** Effects of ketamine treatments on monitored body weight, survival rate, food intake, and water intake across the entire study post cachexia induction with 5-fluorouracil.

Category	Statistics	Control Saline	0 mg/kg (Mock)	15 mg/kg (Low Dose)	30 mg/kg (High Dose)	Test Statistics	*p*-Value GLM
Body weight Baseline At Day 10	N Mean (SD) Min to Max Median Q1, Q3	4 21.9 (0.6) 21.3 to 22.6 21.9 21.4, 22.4	4 22.7 (0.5) 22.2 to 23.3 22.7 22.4, 23.0	4 22.1 (0.9) 21.1 to 23.1 22.1 21.3, 22.9	4 22.1 (0.7) 21.1 to 22.7 22.3 21.6, 22.5	1.0559	0.4038
Body Weight At day 17	N Mean (SD) Min to Max Median Q1, Q3	4 21.0 (0.6) 20.6 to 21.9 20.9 20.7, 21.4	4 18.8 (1.4) 17.3 to 20.8 18.5 17.8, 19.7	4 18.3 (3.8) 14.2 to 23.0 18.0 15.4, 21.2	4 20.3 (1.8) 18.2 to 22.1 20.5 18.8, 21.8	1.3207	0.4038
Body Weight Change from Baseline At day 17	N Mean (SD) Min to Max Median Q1, Q3	4 −0.8 (0.6) −1.7 to −0.4 −0.6 −1.3, −0.4	4 −4.0 (1.3) −4.9 to −2.0 −4.5 −4.8, −3.2	4 −3.8 (2.9) −6.9 to −0.0 −4.2 −6.0, −1.7	4 −1.8 (1.3) −2.9 to −0.3 −2 −2.8, −0.7	3.0771	0.3113
Body Weight % Change from Baseline At day 17	N Mean (SD) Min to Max Median Q1, Q3	4 −3.8 (2.7) −7.4 to −1.7 −3 −5.9, −1.7	4 −17.4 (6.0) −21.9 to −8.6 −19.6 −21.0, −13.9	4 −17.7 (13.8) −32.8 to −0.2 −18.9 −28.0, −7.4	4 −8.2 (5.9) −13.7 to −1.2 −8.8 −13.0, −3.3	2.8414	0.0685
Survival Rate	Rate	4/4 (100%)	1/4 (25%)	1/4 (25%)	4/4 (100%)		
Food Intake (gm)	N Mean (SD) Min to Max Median Q1, Q3	13 18.1 (3.7) 12.0 to 24.1 18.4 15.1 to 20.8	13 14.6 (7.5) 6.0 to 36.6 13.2 10.3 to 16.7	13 16.5 (8.4) 5.3 to 36.3 14.81 11.9 to 19.8	13 16.8 (5.7) 7.8 to 28.6 15.7 13.4 to 19.9	2.4554	0.0876
Water Intake (mL)	N Mean (SD) Min to Max Median Q1, Q3	13 15.4 (3.0) 10.8 to 20.3 15.9 12.6 to 17.8	13 11.5 (3.3) 6.4 to 16.6 11.2 8.7 to 14.0	13 10.8 (3.9) 5.8 to 17.1 12.0 7.2 to 13.4	13 14.5 (3.0) 7.7 to 19.0 15.2 12.9 to 17.0	8.5650	0.0005

## Data Availability

The data presented in this study are available on request from the corresponding authors.
